# Digital versus radiographic accuracy evaluation of guided implant surgery: an in vitro study

**DOI:** 10.1186/s12903-022-02585-5

**Published:** 2022-11-24

**Authors:** Chun Yi, Sha Li, Aonan Wen, Yong Wang, Yijiao Zhao, Yu Zhang

**Affiliations:** 1 Department of Oral Implantology, Peking University School and Hospital of Stomatology & National Clinical Research Center for Oral Diseases & National Engineering Research Center of Oral Biomaterials and Digital Medical Devices & Beijing Key Laboratory of Digital Stomatology, 22 Zhongguancun South Avenue, Beijing, 100081 People’s Republic of China; 2 Center of Digital Dentistry, Peking University School and Hospital of Stomatology & National Clinical Research Center for Oral Diseases & National Engineering Research Center of Oral Biomaterials and Digital Medical Devices & Beijing Key Laboratory of Digital Stomatology, 22 Zhongguancun South Avenue, Beijing, 100081 People’s Republic of China

**Keywords:** Dental implant, Guided surgery, Accuracy, Digital registration, Cone-beam computed tomography

## Abstract

**Background:**

Cone-beam computed tomography (CBCT) is the most widely used method for postsurgical evaluation of the accuracy of guided implant surgery. However, the disadvantages of CBCT include radiation exposure, artifacts caused by metal implants, and high cost. Few studies have introduced a digital registration method to replace CBCT for evaluating the accuracy of guided surgery. The purpose of this study was to compare digital registration to conventional CBCT in terms of the capacity to evaluate the implant positioning accuracy of guided surgery.

**Materials and methods:**

This in vitro study included 40 acrylic resin models with posterior single mandibular tooth loss. Guided surgery software was used to determine the optimal implant position; 40 tooth-supported fully guided drilling templates were designed and milled accordingly. After the guided surgery, the accuracies of the surgical templates were evaluated by conventional CBCT and digital registration. For evaluation by conventional CBCT, postsurgical CBCT scans of the resin models were performed. The CBCT data were reconstructed and superimposed on the implant planning data. For digital registration, we constructed a virtual registration unit that consisted of an implant replica and a scan body. Next, we obtained postsurgical optical scans of resin models with the scan body. The postsurgical implant position was identified by superimposition of the registration unit and optical scan data. The implant planning data and postsurgical implant position data were superimposed; deviations were reported in terms of distance for implant entry/apex point and in terms of angle for the implant axis. Interclass correlation coefficients (ICCs) and Bland–Altman plots were used to analyze the agreement between the two evaluation methods.

**Results:**

The ICCs between the two methods were 0.986, 0.993, and 0.968 for the entry point, apex point, and angle, respectively; all were significantly greater than 0.75 (*p* < 0.001). Bland–Altman plots showed that the 95% limits of agreement of the differences were − 0.144 to + 0.081 mm, − 0.135 to + 0.147 mm, and − 0.451° to + 0.729° for the entry point, apex point, and angle, respectively; all values were within the maximum tolerated difference.

**Conclusion:**

Conventional CBCT and digital registration showed good agreement in terms of evaluating the accuracy of implant positioning using tooth-supported surgical templates.

## Background

Implant-supported prostheses are useful for replacing missing teeth [1]. Appropriate three-dimensional (3D) positioning of dental implants is important for long-term implant stability and good aesthetic outcomes [2]. Surgical templates are commonly used to apply implants in the presurgically planned position to ensure adequate space for future prosthetic insertion [3]. Surgical templates can be designed by incorporating the superimposed cone-beam computed tomography (CBCT) data and optical surface scanning data into the guided surgery software [4, 5]. Many previous studies have shown that the use of surgical templates can reduce discrepancies between planned and actual implant positions, compared with freehand implant placement [2, 3, 6–8]. Tahmaseb et al. [2] performed a systematic review of 24 studies of the accuracy of partially edentulous tooth-supported surgical guides; they reported mean deviations of 0.84 mm, 1.15 mm, and 3.28° at the implant entry point, apex point, and angulation, respectively.

Postsurgical CBCT can be used to confirm proper positioning of the inserted dental implant in the jawbone; it can also be used to evaluate the accuracy of guided implant surgery involving surgical templates and implant navigation systems [9–14]. Many dental digital software can be used to evaluate the discrepancies between planned and actual implant positions via superimposition of planning digital data and postsurgical CBCT data. However, two CBCT scans must be performed for each patient——one before and the other one after the guided surgery. Since the increased radiation exposure and the additional biological cost of CBCT, a postsurgical CBCT examination to evaluate the implant position should not be considered as a clinical necessity and in larger cohorts is limited for ethical reasons [15, 16]. Up till now conventional two-dimensional (2D) X-ray image is still the prime tool for postsurgical implant monitoring [17]. Jacobs et al. [18] recommended the use of postsurgical CBCT to evaluate graft healing and complications related to neurovascular trauma; this assessment could also be used to plan implant removal in cases of infection or mechanical failure. However, CBCT is not appropriate for routine follow-up after implant surgery [16]. Furthermore, the accuracy of CBCT image superimposition is greatly influenced by image quality, which is affected by voxel size, metal implant-related artifacts, patient movement, and scanning parameters [14, 19, 20]. Previous studies have extensively evaluated the usefulness of radiographic evaluation for determining the accuracy of implant positioning after guided surgery; however, its usefulness is limited in cases with no indications for postsurgical CBCT [15, 16].

To overcome the disadvantages of postsurgical CBCT, a number of non-radiographic methods were recently developed to conduct 3D analysis of planned and actual implant positions [21–26]. The frequently-used non-radiographic method involves digital registration in which the planned implant position (determined by the guided surgery software) is matched with the actual implant position (detected by optical scanning). Derksen et al. [21] evaluated the accuracy of guided implant surgery by performing postsurgical digital impression using an optical scanner after connecting a scan body to the inserted implant, followed by the creation of an open source Standard Tessellation Language (STL) file that could be imported into the guided surgery software (coDiagnostiX; Dental Wings GmbH, Chemnitz, Germany). After the registration procedure had been applied using the “Treatment Evaluation Tool” included in the software, the postsurgical intraoral scan data and implant analog location were matched to the presurgical implant planning data. The software automatically analyzed the discrepancies between the planned and actual implant positions. This digital evaluation method eliminates the need for postsurgical CBCT, thereby preventing unnecessary radiation exposure. Furthermore, postsurgical optical scanning can avoid the effects of CBCT quality on data registration accuracy [27]. There were few previous in vitro studies reported the deviations between implant positions determined by a surface scanner and by postsurgical CBCT [28, 29]. Derksen et al. [21] suggested the need for more studies to confirm that the results of the digital registration method are similar to the results of conventional postsurgical CBCT in terms of assessing the accuracy of guided surgery.

In the present in vitro study, we compared digital registration to conventional CBCT in terms of the capacity to evaluate the accuracy of implant positioning. The null hypothesis (H0) was that there was no difference between digital registration and conventional CBCT in terms of the capacity to evaluate the accuracy of implant positioning after guided surgery.

## Materials and methods

### Sample size calculation

The present in vitro study evaluated the agreement between two evaluation methods (digital registration and conventional CBCT) for assessing the accuracy of guided implant surgery. We calculated interclass correlation coefficients (ICCs) to confirm whether the accuracy of the digital registration method was equivalent to the accuracy of the conventional CBCT method [30, 31]. Analysis in PASS software (version 15; NCSS, LLC., Kaysville, Utah, USA) showed that 36 samples per group were necessary to achieve a power of 90% (β = 0.10) for detecting an ICC of 0.90 under the alternative hypothesis, when the ICC under the null hypothesis was 0.75 and the significance level was 0.05 (α = 0.05). To allow for potential dropout rate, we included 40 models in each group.

### Presurgical planning and surgery

This in vitro study included 40 acrylic resin models with posterior single mandibular tooth loss that were used for undergraduate education. The fully guided implant surgeries were performed by 40 final year dental students from Peking University School and Hospital of Stomatology. The in vitro study protocol followed the CRIS reporting guidelines.

**Fig. 1:**
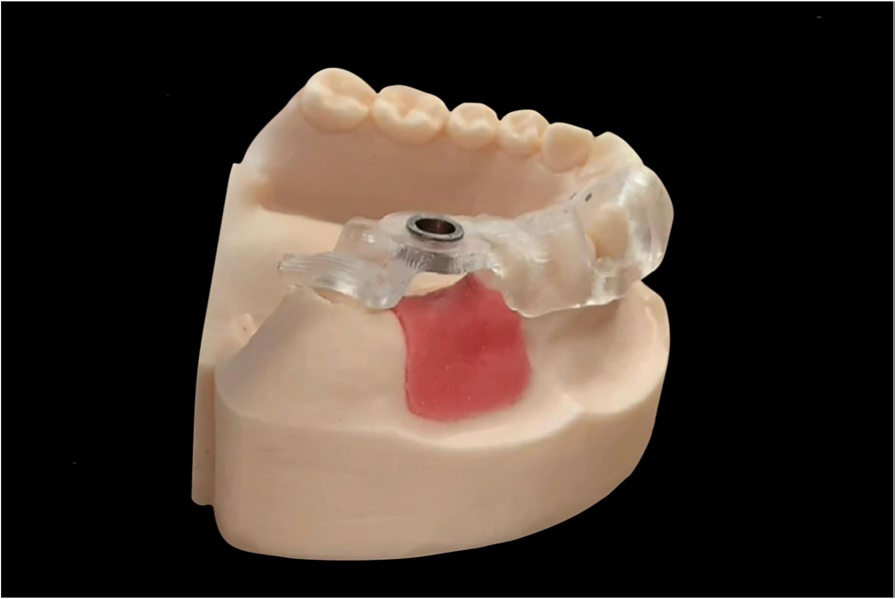
Fully guided template and acrylic resin model

Design and fabrication of the fully guided template and the acrylic resin model (Fig. 1):

A 35-year-old female patient requested dental implantation for a missing right mandibular first molar at Peking University School and Hospital of Stomatology, Department of Oral Implantology. She was otherwise healthy and had no contraindications to implant surgery. The patient provided written informed consent for the use of her data for teaching and research purposes. All procedures related to the human participant were conducted in accordance with the 1975 Declaration of Helsinki revised in 2000 and approved by the local ethics committee (Institutional Review Board of Peking University School and Hospital of Stomatology; Approval Numbers: PKUSSIRB-201736075).

The patient underwent CBCT using a Planmeca ProMax™ 3D scanner (Planmeca Oy, Helsinki, Finland). The 3D CBCT data were exported as a Digital Imaging and Communications in Medicine (DICOM) file. The following standardized projection settings were used: field-of-view (FOV) diameter, 10 cm; FOV height, 5.6 cm; acceleration voltage, 90 kV; beam currency, 8.0 mA; and voxel size, 0.2 mm. An impression of the patient's mandibular teeth was obtained using silicone impression material (Silagum-Light and Silagum-MixStar Putty Soft; DMG Medical Devices, Rome, Italy). A gypsum cast (Modern Materials, Die-Stone; Kulzer GmbH, Hanau, Germany) was poured and used as the master model. The master model was optically scanned three times using a highly accurate dental laboratory scanner (3Shape E4; 3Shape, Copenhagen, Denmark) to confirm its reproducibility. The 3D data of the master model were exported in STL format. The 40 mandibular acrylic resin models used in subsequent surgeries were fabricated by the 3D printer (AccuFab-C1s; SHINING 3D, Hangzhou, China) from a dental laboratory according to the 3D data of the master model.

The CBCT data (DICOM format) and optical scanning data of the master model (STL format) were imported into the implant planning software (Simplant, v11.04; Dentsply Sirona, Ballaigues, Switzerland) to determine the ideal implant position. For this purpose, the optical scanning data were aligned with the CBCT data, and a prosthetic-driven virtual set-up was created. The implant position was determined by the virtual prosthesis and anatomical structures. After the implant position had been planned, a tooth-supported fully guided drilling template was designed and sent to the milling unit (CEREC MC XL Premium; Dentsply Sirona) for the milling of 40 templates (CEREC Guide Bloc medi; Dentsply Sirona). After cleaning and polishing of the templates, titanium sleeves were positioned into the drilling templates.

**Fig. 2:**
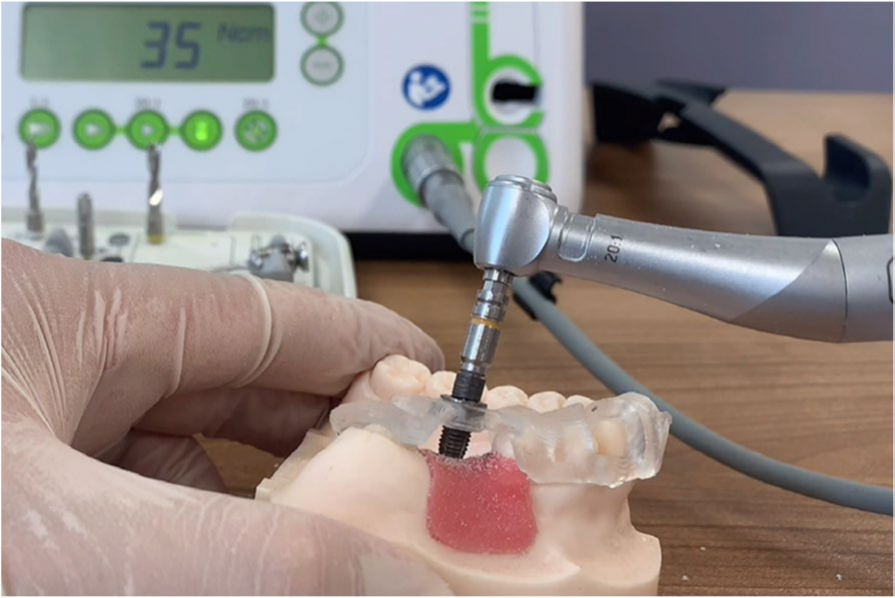
Fully guided implant surgery on the resin model

Surgical procedure (Fig. 2):

Before the surgery, adequate seating of the tooth‐supported drilling templates on the resin models were confirmed. Implant surgeries were performed on the 40 acrylic resin models by 40 final year dental students who had completed their theoretical education related to implant placement. Each surgery was performed by two dental students and supervised by a maxillofacial surgeon, in accordance with the manufacturer's instructions regarding the guided surgery drill sequence. The artificial gingiva was punched out and removed. Then, the implant bed was prepared and the implant (4.2 × 13 mm; Astra Tech Implant System® OsseoSpeed® EV, Dentsply Sirona) was inserted with the drilling templates in situ.

### Methods to acquire postsurgical implant position and to evaluate accuracy of guided implant surgery

#### Conventional radiographic method

In the conventional radiographic method, postsurgical CBCT was performed to determine the implant position for all acrylic resin models with inserted implants but without any superstructures, such as abutments or prostheses. Postsurgical CBCT images were acquired using a Planmeca ProMax™ 3D scanner (Planmeca Oy). The technical parameters, which differed from the parameters used for presurgical CBCT, were as follows: FOV diameter, 10 cm; FOV height, 5.6 cm; acceleration voltage, 66 kV; beam currency, 1.0 mA; voxel size, 0.2 mm; and metal artifact reduction mode.

**Fig. 3:**
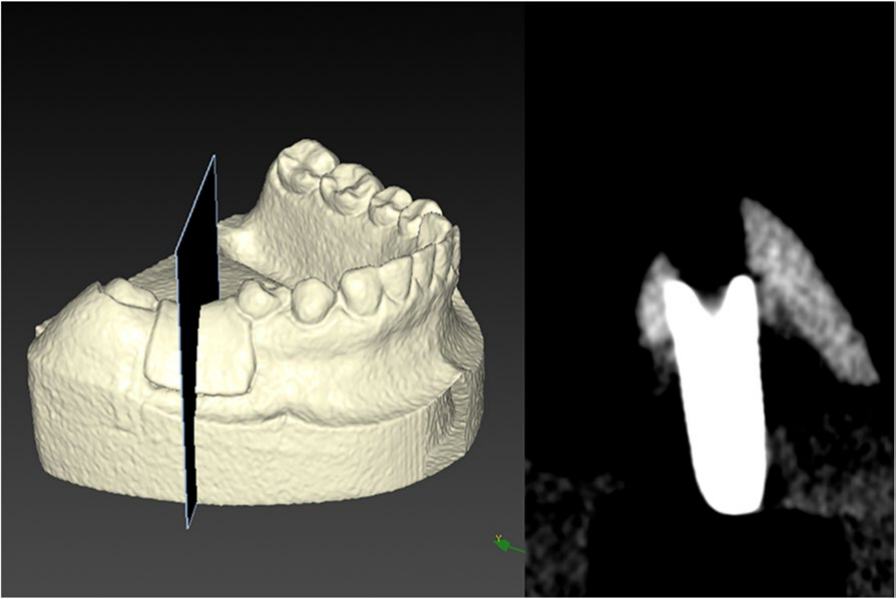
3D reconstruction of postsurgical CBCT (left; STL-CBCT) and its 2D cross-section of implant (right)

The CBCT data (DICOM format) were transferred to volumetric imaging software (Mimics 15.0; Materialise, Leuven, Belgium), in which the virtual acrylic resin mandibular models and inserted implants were subjected to 3D reconstruction, then saved in STL format (STL-CBCT; Fig. 3). The postsurgical implant and entire model were reconstructed separately based on their Hounsfield unit values, using a common coordinate system.

**Fig. 4:**
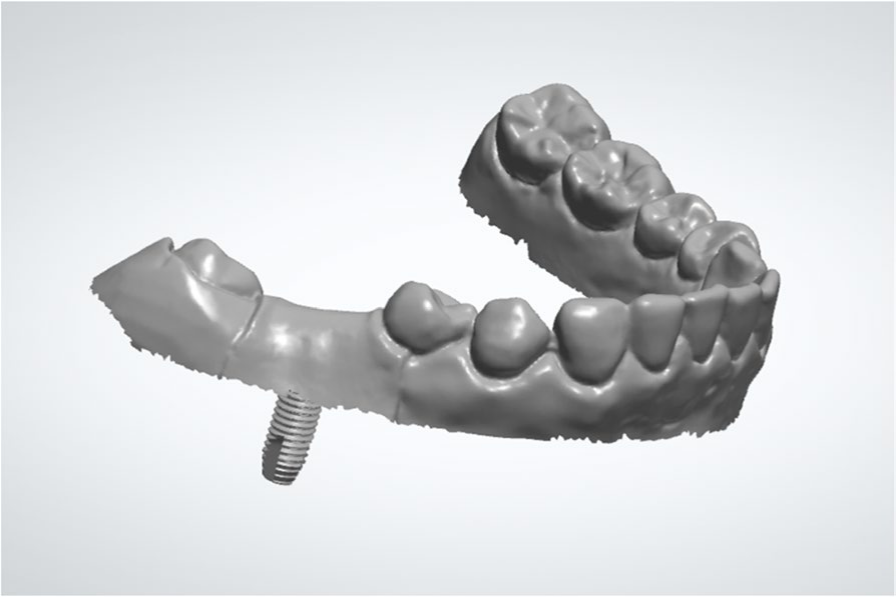
Planned implant position (STL-PLAN)

Data regarding the planned 3D implant position and the presurgical virtual 3D model were exported from the implant planning software (Simplant, v11.04; Dentsply Sirona) as the planning digital data in STL format (STL-PLAN; Fig. 4).

**Fig. 5:**
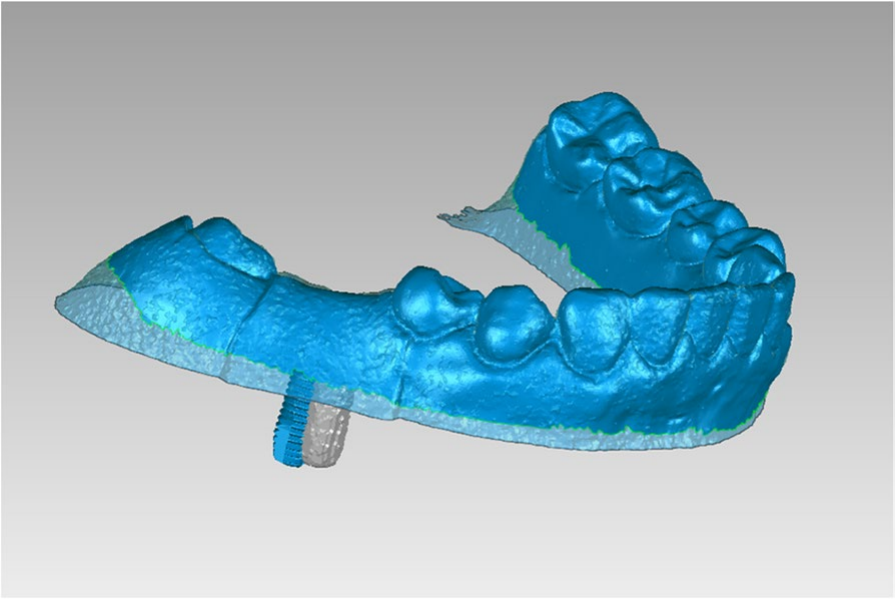
The STL files obtained from the presurgical plan and the postsurgical CBCT reconstruction were superimposed

The STL-PLAN and STL-CBCT files were superimposed based on the data of the overall dentition using the “best-fit alignment” function in reverse engineering software (Geomagic Studio 2014; Geomagic, 3D Systems, Rock Hill, SC, USA) (Fig. 5).

**Fig. 6:**
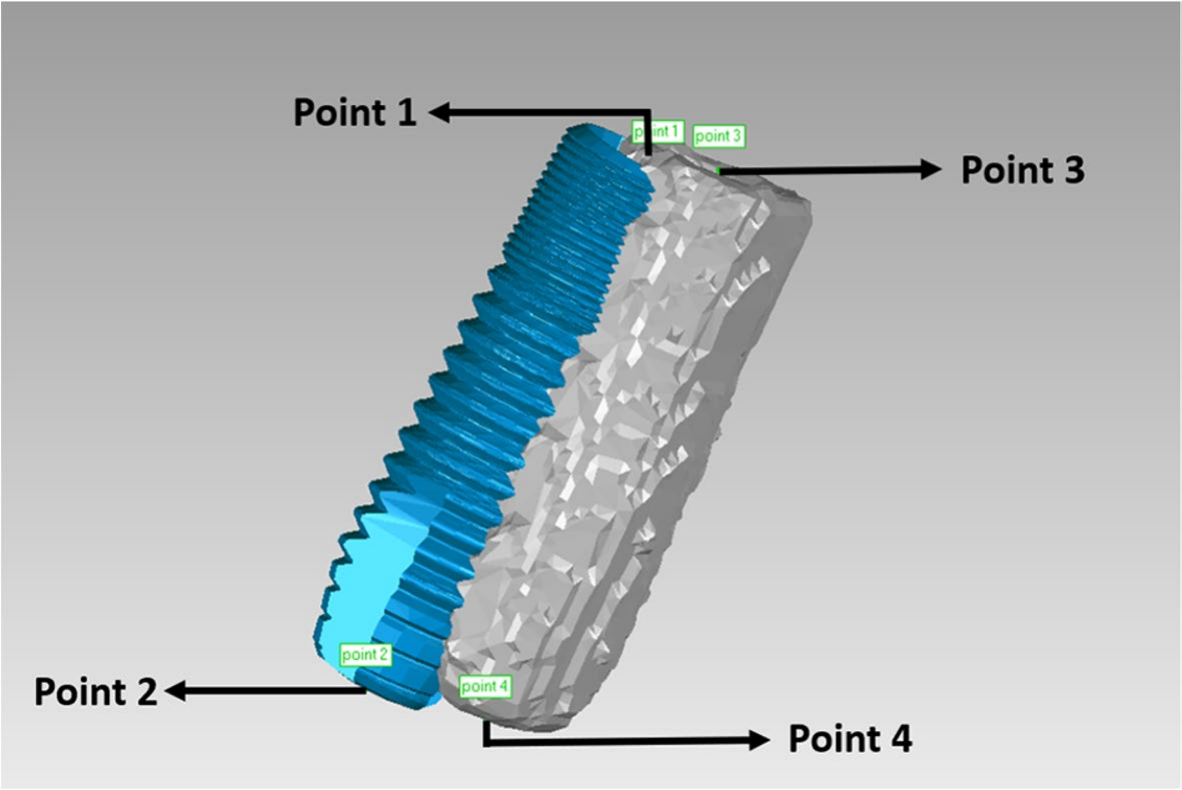
The positional relationship between the planned (blue) and actual (grey) implant sites was determined by the conventional radiographic method. (Point 1: the entry point of the planned implant; Point 2: the apex point of the planned implant; Point 3: the entry point of the actual implant determined by the conventional radiographic method; Point 4: the apex point of the actual implant determined by the conventional radiographic method)

**Fig. 7:**
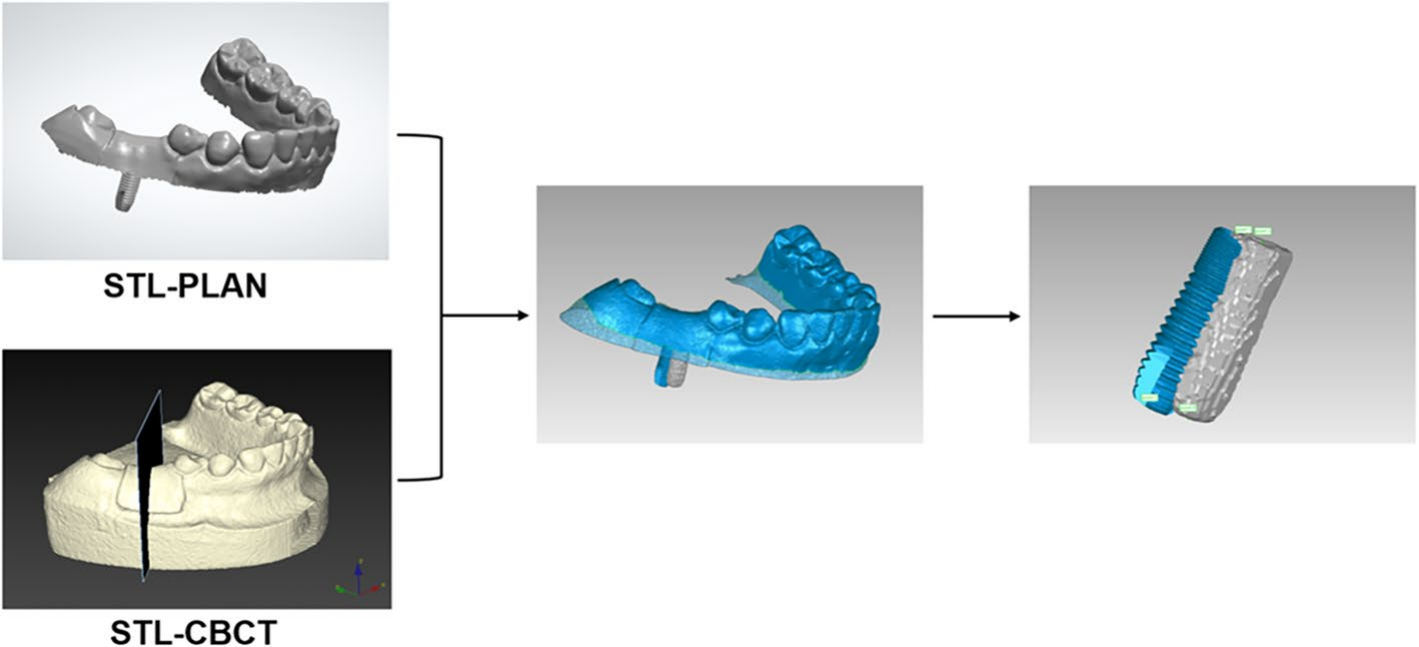
Flowchart of the conventional radiographic method

After the superimposed STL files had been trimmed, the positional relationship between the planned and actual implant sites was determined (Fig. 6). To make a measurement, the implant entry and apex points were labeled using the “rotation axis” function in Geomagic software (Geomagic Studio 2014; Geomagic, 3D Systems). The rotation axis was automatically fitted by the software according to the contour of the implant. The intersection of the rotation axis and the implant cervix/bottom was regarded as the entry/apex point. Points 1 and 2 were defined as the entry and apex points of the planned implant, respectively. Points 3 and 4 were defined as the entry and apex points of the actual implant, as determined by the conventional radiographic method. Accuracy was evaluated using three outcomes: linear distance deviations (mm) between the planned and actual implants at the entry point (distance between points 1 and 3) and apex point (distance between points 2 and 4), and the angular deviation (°) between implant axes. Figure 7 presents a flowchart of the conventional radiographic method.

#### Digital registration method

**Fig. 8:**
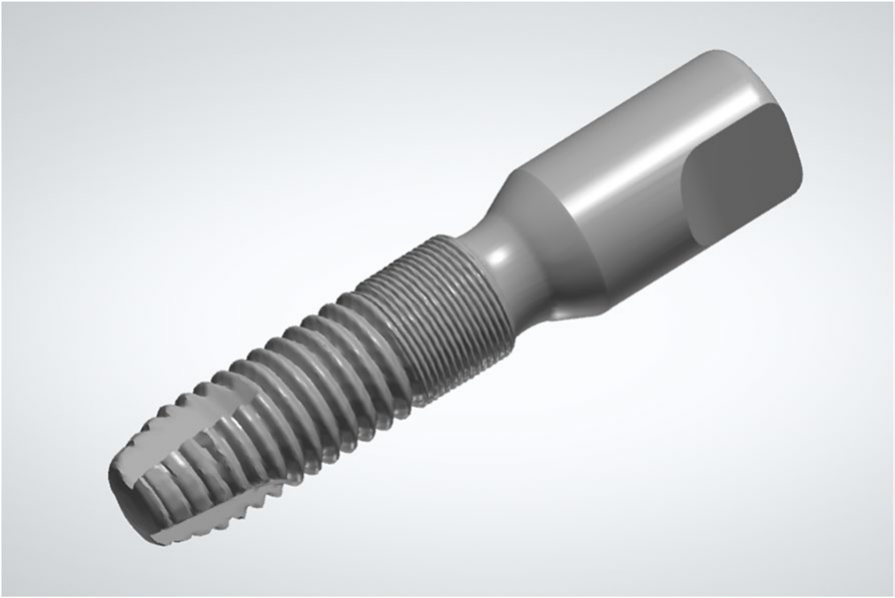
Construct a virtual registration unit that composed of an implant replica and a scan body (STL-REGISTRATION UNIT)

To determine the postsurgical implant position using the digital registration method, the implant used in the surgery (4.2 × 13 mm; Astra Tech Implant System® OsseoSpeed® EV, Dentsply Sirona) was connected with a compatible scan body (AE42-SB; TruAbutment, Irvine, CA, USA). This integrated component defined as a registration unit was scanned by a lab scanner (3Shape E4, 3Shape), and the 3D model of the registration unit was reconstructed based on reverse engineering and then saved in STL format (STL-REGISTRATION UNIT; Fig. 8).

**Fig. 9:**
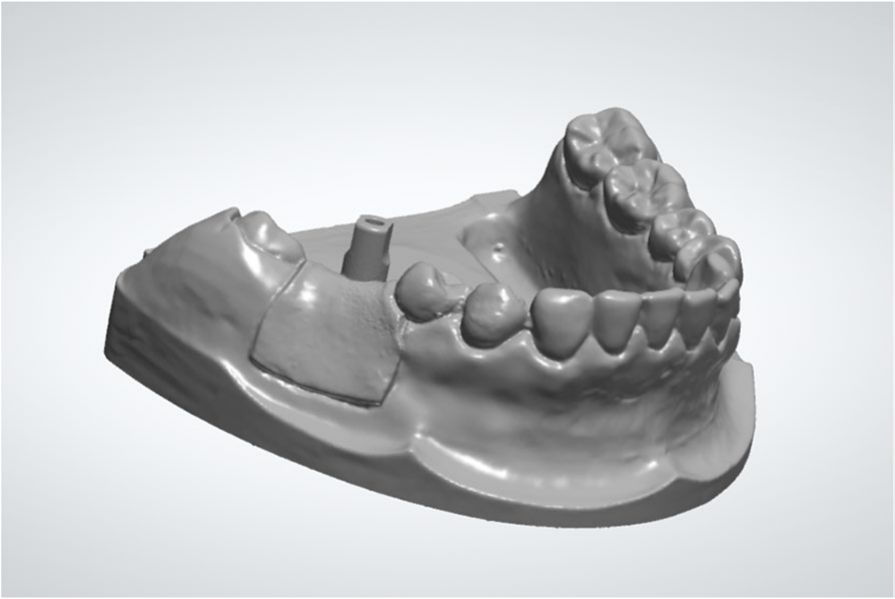
Take postsurgical optical scan of the resin model with the scan body (STL-MODEL)

The scan body (AE42-SB; TruAbutment) was connected to the implant inserted in the acrylic resin model. The postsurgical model was optically scanned by an experienced operator using the lab scanner described above (3Shape E4; 3Shape). The optical scanning data of the model was saved in STL format (STL-MODEL; Fig. 9).

**Fig. 10:**
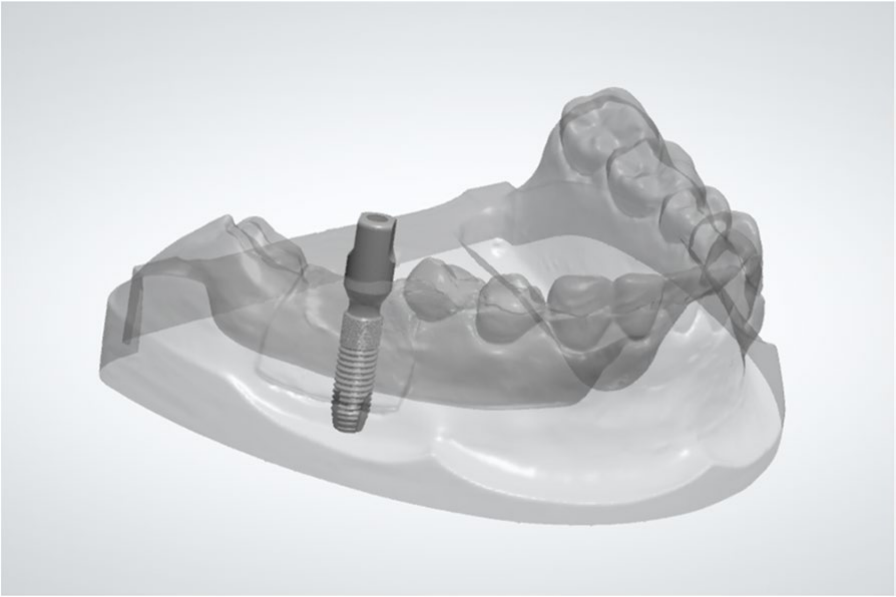
Obtain the postsurgical implant position by superimposing the registration unit data and the optical scan data (STL-MODEL & IMPLANT)

To identify the postsurgical implant position, STL-REGISTRATION UNIT and STL-MODEL files were imported into reverse engineering software (Geomagic Studio 2014; Geomagic, 3D Systems) and superimposed using the “best-fit alignment” function with reference to the scan body data, which was regarded as the common region within the two STL files. The postsurgical implant position obtained by digital registration was exported in STL format (STL-MODEL & IMPLANT; Fig. 10).

**Fig. 11:**
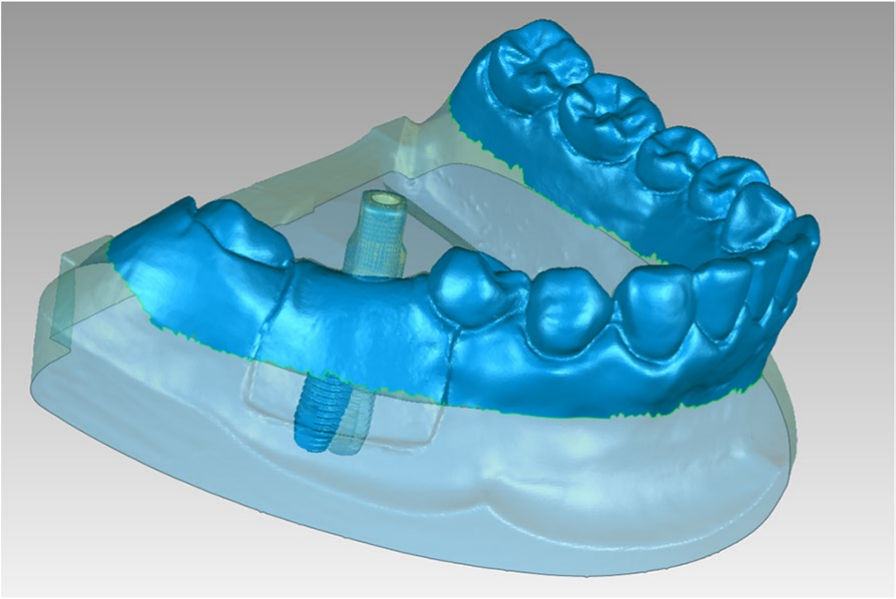
Obtain the positional relationship between the planned and actual implant by superimposing the planned and postsurgical implant position data

The planned implant position data (STL-PLAN) and postsurgical implant position data (STL-MODEL & IMPLANT) were imported into Geomagic software (Geomagic Studio 2014; Geomagic, 3D Systems) and aligned using the “best-fit alignment” function, according to the corresponding sites of dentition (Fig. 11).

**Fig. 12:**
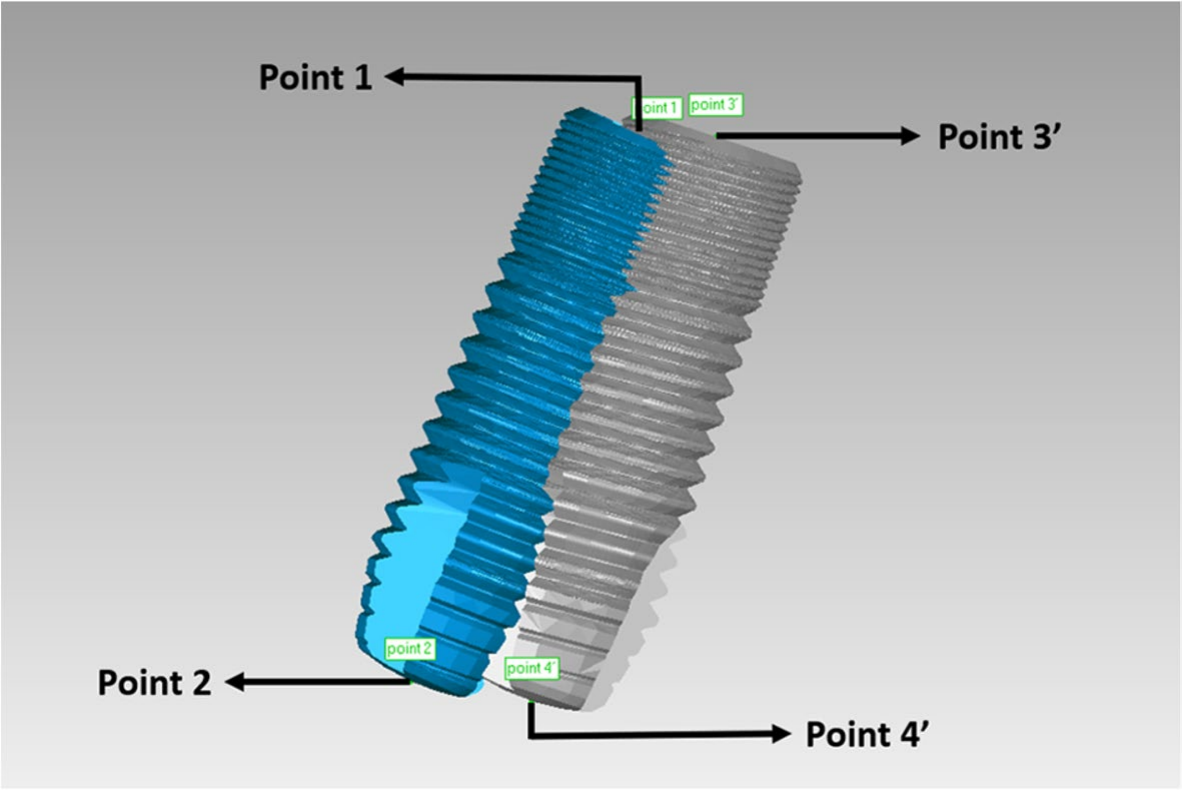
The positional relationship between the planned (blue) and actual (grey) implant sites was determined by the digital registration method. (Point 1: the entry point of the planned implant; Point 2: the apex point of the planned implant; Point 3’: the entry point of the actual implant determined by the digital registration method; Point 4’: the apex point of the actual implant determined by the digital registration method)

**Fig. 13:**
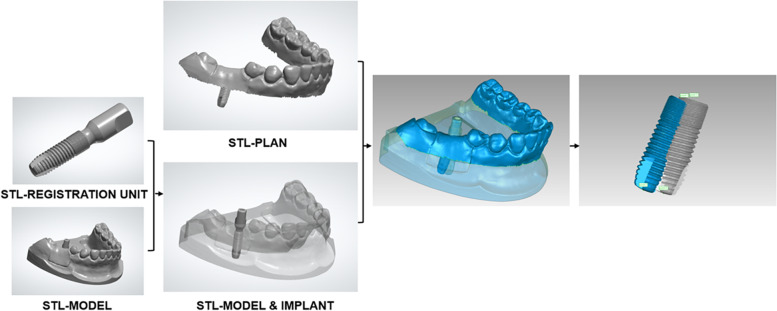
Flowchart of the digital registration method

By deleting the redundant parts such as dentition and gingiva in the planning and postsurgical digital data using the “selecting bounded components” function, and then trimming the part of the scan body using the “trimming with a plane” function in Geomagic software (Geomagic Studio 2014; Geomagic, 3D Systems), the positional relationship between the planned and actual implant sites remained. After a measurement procedure had been applied as described above for the conventional radiographic method, we measured the relative positions of the planned and actual implants (Fig. 12); including the angular deviation between the two implant axes, and the linear distance deviations between the two implant entry points (distance between points 1 and 3’) and the two apex points (distance between points 2 and 4’). Points 3’ and 4’ were defined as the respective entry and apex points of the actual implant, as determined by the digital registration method. Figure 13 presents a flowchart of the digital registration method.

#### Statistical analysis

Descriptive analyses were performed for all variables. The accuracy of guided implant surgery, in terms of angular deviation of the axis and linear distance deviations of the implant entry and apex points, was evaluated using digital registration and conventional radiographic methods. Scatter plots were constructed to show correlations between the two methods.

ICCs were used to analyze the agreement between the two methods in terms of assessing the accuracy of guided implant surgery. ICCs > 0.75 were considered indicative of good agreement between the digital registration and conventional radiographic methods [30].

**Fig. 14:**
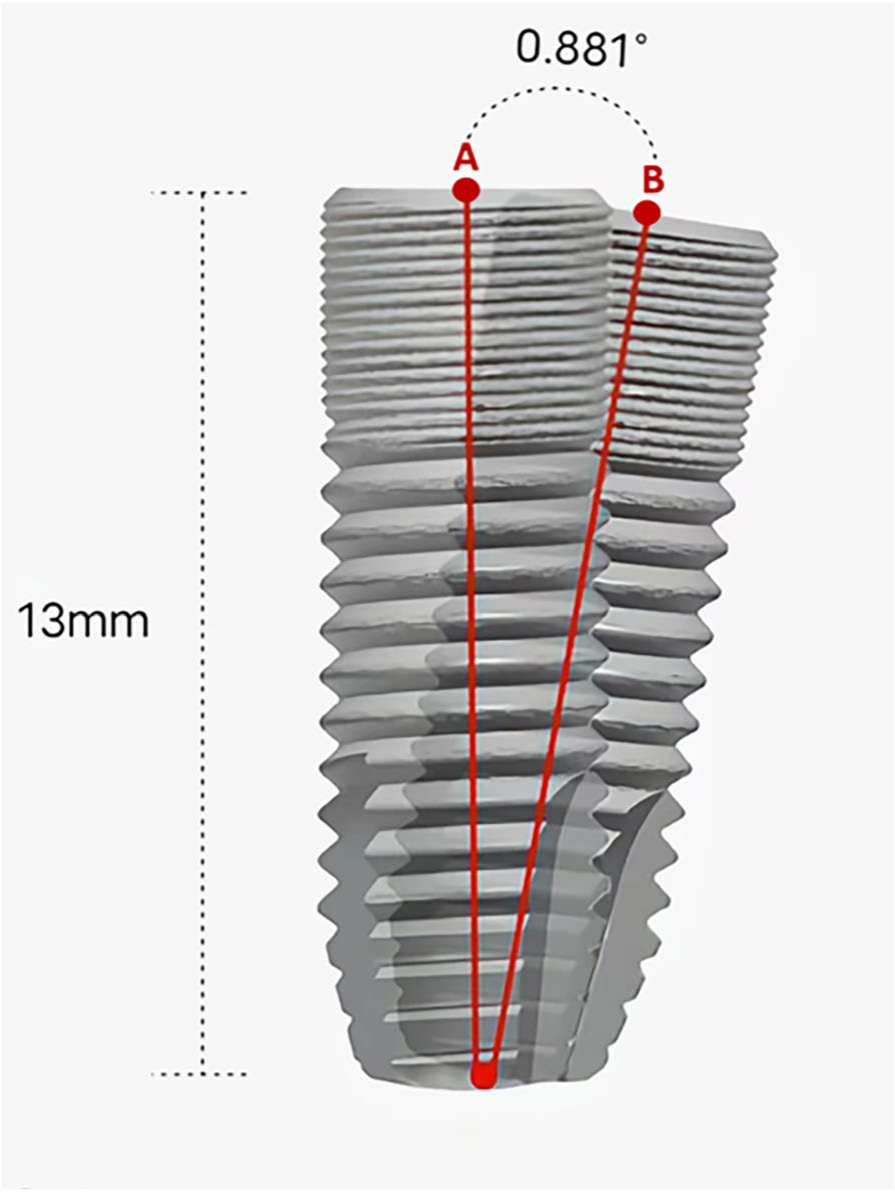
Diagrammatic sketch of maximum tolerated difference. Based on the findings in previous studies [19, 24] and the resolution of CBCT (voxel size: 0.200 mm), we used a maximum tolerated difference of 0.200 mm for linear distance deviation at the entry or apex points (the distance between point “A” and “B”). A maximum tolerated difference of 0.881°(360° × 0.2 mm / 2π × 13 mm) for the angular deviation of two implants’ axes was calculated according to the length of the implant (13 mm) and the distance between point “A” and “B” (0.200 mm)

Bland–Altman plots were used to analyze the difference scores between the two methods and the mean score for each individual method, then determine agreement between the methods. If the 95% difference value was within the 95% limits of agreement (LoA) or the LoA was within the maximum tolerated difference in the Bland–Altman plots, there was good agreement between the two methods [32]. Based on the findings in previous studies [20, 27] and the resolution of CBCT (voxel size: 0.2 mm), we used a maximum tolerated difference of 0.200 mm for linear distance deviation at the entry and apex points, along with a maximum tolerated difference of 0.881° (360° × 0.2 mm / 2π × 13 mm) for the angular deviation of the axis. (Fig. 14).

SPSS software (version 26; IBM Corp., Armonk, NY, USA) was used to analyze the descriptive statistics, construct scatter plots, and perform ICC analyses. Bland–Altman plots were constructed using MedCalc software (version 19.3; MedCalc Software Ltd., Belgium).

## Results

**Fig. 15:**
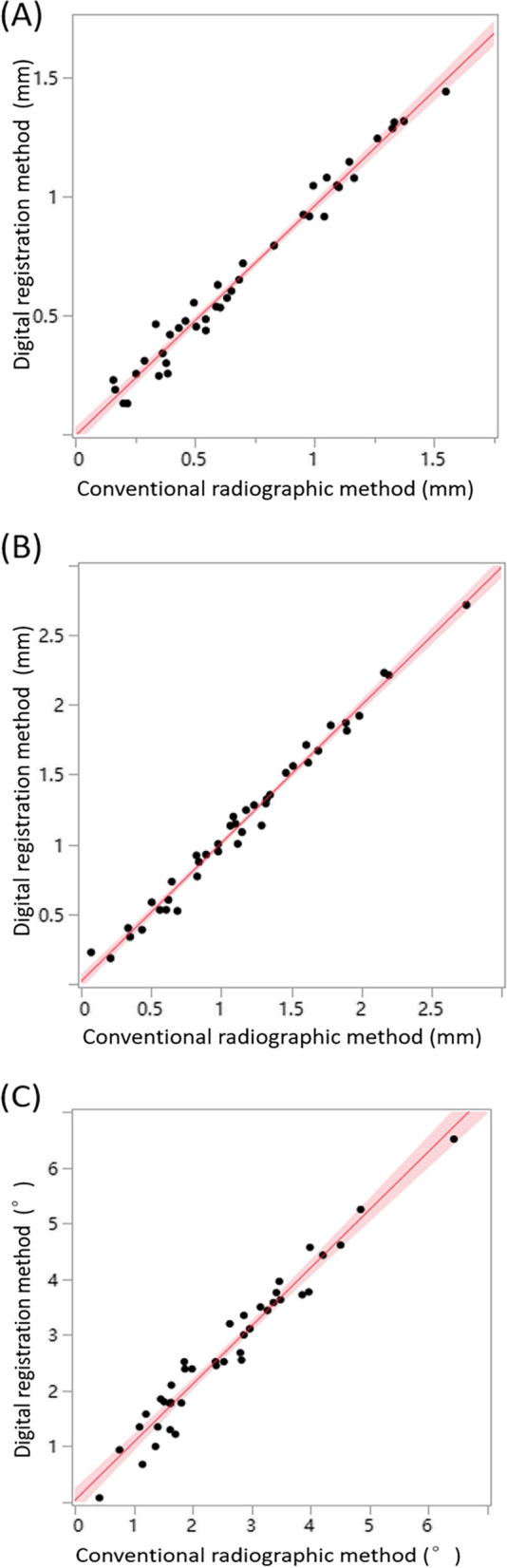
The scatter diagrams of (**A**) linear distance deviation at the entry point, (**B**) linear distance deviation at the apex point and (**C**) angular deviation of the axis. The x- and y-axes represent the deviations between the planned and actual implants identified by the conventional radiographic and digital registration methods, respectively

In this in vitro study, 40 implants were inserted into 40 acrylic resin mandible models fully guided by 40 tooth-supported surgical templates. The accuracy of each surgical template was evaluated by both conventional CBCT and digital registration. Table 1 presents the analysis of guided implant surgery accuracy, as determined by the two methods. According to the conventional radiographic method, the mean deviations of the actual implant position from the planned implant position were 0.704 ± 0.388 mm, 1.154 ± 0.601 mm, and 2.561° ± 1.259° for the entry point, apex point, and angle, respectively. According to the digital registration method, the mean deviations of the actual implant position from the planned implant position were 0.672 ± 0.379 mm, 1.160 ± 0.598 mm, and 2.700° ± 1.345° for the entry point, apex point, and angle, respectively. Fig. 15 presents scatter plots of the correlation between the two methods; its x- and y-axes represent the deviations between the planned and actual implants identified by the conventional radiographic and digital registration methods, respectively. The scatter plot shows a straight line, suggesting a linear correlation between the two methods.

**Table 1:** Accuracy of guided implant surgery determined by the conventional radiographic method and the digital registration method

Group		Entry Point (mm)	Apex Point (mm)	Angle (°)
Conventional radiographic method	Mean	0.704	1.154	2.561
	SD	0.388	0.601	1.259
	95% CI	0.580–0.828	0.962–1.346	2.158–2.964
Digital registration method	Mean	0.672	1.160	2.700
	SD	0.379	0.598	1.345
	95% CI	0.551–0.793	0.969–1.351	2.270–3.130

*SD* Standard deviation, *CI* Confidence interval

“Entry Point”, “Apex Point” and “Angle” refer to the deviations of the actual implant position from the planned implant position for the entry point, apex point, and angle, respectively

**Fig. 16:**
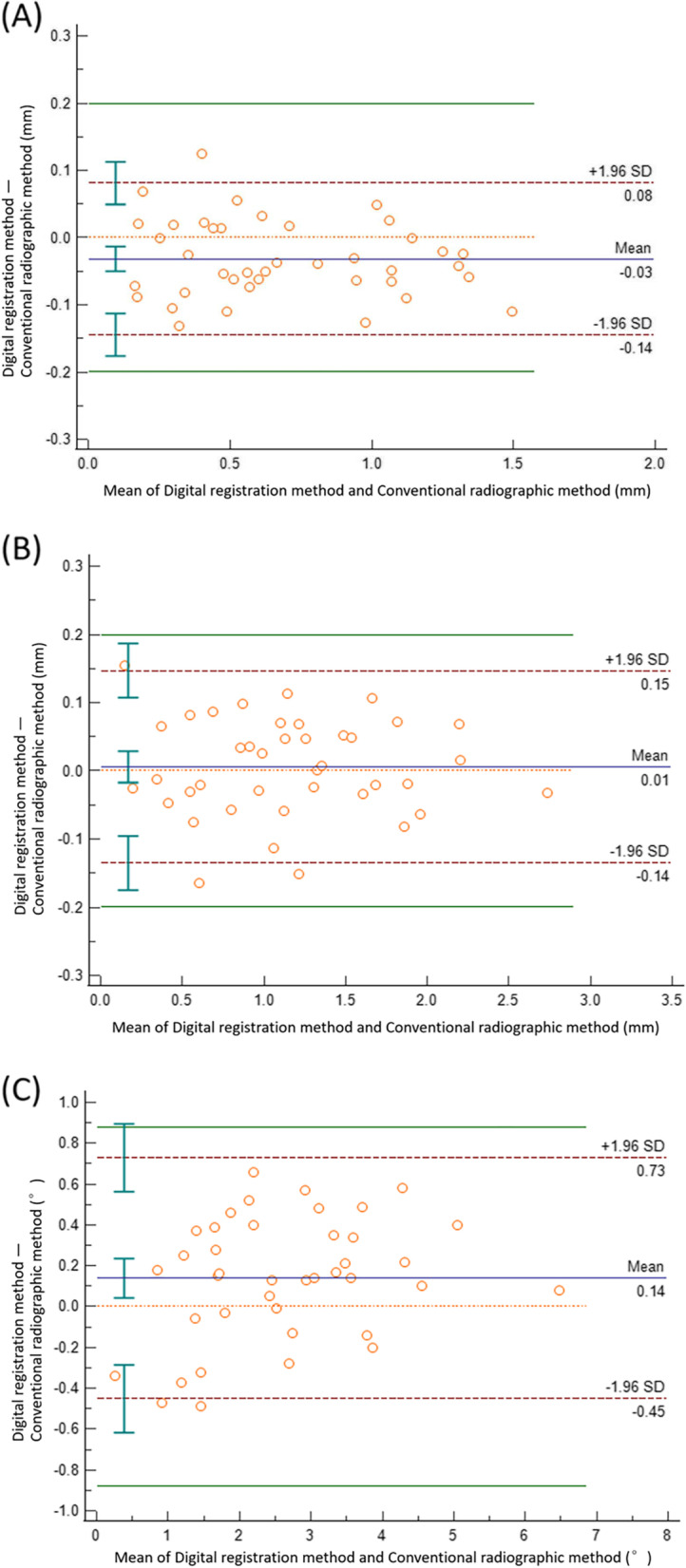
The Bland–Altman plots of (**A**) linear distance deviation at the entry point, (**B**) linear distance deviation at the apex point and (**C**) angular deviation of the axis between the planned and actual implants identified by the conventional radiographic and digital registration methods. Bland–Altman plots include the mean difference (blue), the limits of agreement (red dotted line) with an approximate 95% CI of the limits of the agreement, and the maximum tolerated difference (green line)

We assessed the agreement between the two methods using ICC and Bland–Altman plots. The ICCs between the digital registration and conventional radiographic methods for the entry point, apex point, and angle were 0.986, 0.993, and 0.968, respectively; all values were significantly greater than 0.75 (*p* < 0.001), which indicated good agreement between the two methods (Table 2). The Bland–Altman plots showed that the mean differences between the two methods were − 0.032 mm, 0.006 mm, and 0.139° for the entry point, apex point, and angle, respectively (Table 3). The LoAs of the difference value (− 1.96 standard deviation [SD] to + 1.96 SD) for the entry point, apex point, and angle were − 0.144 to + 0.081 mm, − 0.135 to + 0.147 mm, and − 0.451° to + 0.729°, respectively; all values were within the range of maximum tolerated difference (− 0.200 to + 0.200 mm and − 0.881° to + 0.881°), which indicated good agreement between the two methods. There were only one difference spot (1/40, 2.5%) out of the LoA for the entry point, two difference spots (2/40, 5%) for the angle, and three difference spots (3/40, 7.5%) for the apex point, indicating that most difference value spots were within the LoAs in the Bland–Altman plots. (Fig. 16).

**Table 2:** ICC statistics between the conventional radiographic method and the digital registration method

	Entry Point	Apex Point	Angle
ICC	0.986	0.993	0.968
95% CI	0.962–0.994	0.987–0.996	0.930–0.985
*P*	< 0.001	< 0.001	< 0.001

*CI* Confidence interval. *P* is calculated by comparing with ICC = 0.75

“Entry Point”, “Apex Point” and “Angle” refer to the deviations of the actual implant position from the planned implant position for the entry point, apex point, and angle, respectively

**Table 3:** Bland–Altman statistics of the conventional radiographic method and the digital registration method

	Entry Point (mm)	Apex Point (mm)	Angle (°)
Mean of difference	-0.032	0.006	0.139
95% limits of agreement	(-0.144, + 0.081)	(-0.135, + 0.147)	(-0.451, + 0.729)
Maximum tolerated difference	(-0.200, + 0.200)	(-0.200, + 0.200)	(-0.881, + 0.881)

Difference = Digital registration method - Conventional radiographic method

“Entry Point”, “Apex Point” and “Angle” refer to the deviations of the actual implant position from the planned implant position for the entry point, apex point, and angle, respectively

## Discussion

In this study, we compared the postsurgical implant positions identified by conventional radiographic and digital registration methods. Our results showed that the two methods had good agreement in terms of evaluating the accuracy of implant positioning using the tooth-supported drill guides. Thus, the null hypothesis (H0) was accepted. Our results indicated that digital registration could be used to evaluate the accuracy of single-tooth implant surgery using tooth-supported drill guides.

The digital registration method was used to investigate the accuracy of guided implant surgery in four steps. First, a virtual registration unit was constructed based on a reverse engineering process. The registration unit was composed of an implant replica and a scan body. Second, a postsurgical optical scan of the dentition was obtained with the scan body. Third, the relative positions of the postsurgical implant and the adjacent dentition were identified in the first registration. The first registration was based on the scan body, which was regarded as the common region of the registration unit and the dentition scan data. Fourth, a second registration was performed, which involved superimposition of the first registration data and planned implant position data according to the corresponding sites of dentition. The 3D positional relationship between the planned and actual implant data was obtained after the superimposed data files had been trimmed.

Several previous studies used CBCT for postsurgical evaluation of 3D implant positioning and for assessments of accuracy concerning surgical templates and implant navigation systems. However, the disadvantages of CBCT include radiation exposure, artifacts caused by metal implants, and high cost [15, 16, 19, 20]. Digital registration avoids the need for postsurgical radiographic examination, thereby reducing radiation exposure and other associated problems (e.g., image distortion, deformation, and artifacts). Comparing with the CBCT scan method, this digital registration method reduces the biological and economic cost for patients [29]. Thus, digital registration is suitable for large-scale clinical research. The accuracy of guided surgery can be evaluated by superimposing the implant planning data and postsurgical data. Postsurgical CBCT data are acquired at a voxel resolution of 0.2 mm; a superimposition precision better than 0.2 mm cannot be achieved using the conventional CBCT method [20, 27]. The manufacturer of the lab scanner reports an accuracy level of 4 μm (3Shape E4, 3Shape, ISO 12836); therefore, the digital registration method has better precision than the conventional CBCT method. In our study, the registration unit and the postsurgical model were optically scanned using the lab scanner (3Shape E4, 3Shape) in the digital registration method. The accuracy of this new method has not been proved by using other types of optical scanners. In addition, using intra-oral scanner instead of lab scanner may reduce the accuracy of this method, because the precision and trueness of intra-oral scanner decrease accordingly when the scanned quadrants increase [33]. But the other study proved that there was no significant difference between the implant positions located via intraoral scanner and extraoral scanner [28].

Our results indicated that the digital registration method could be used to evaluate the accuracy of single-tooth implant surgery. Although tooth-supported drill guides were used in the present study, the digital registration method may also be useful for other single-tooth guided surgeries, such as implant navigation. The digital registration method can also be used for postsurgical assessment of the 3D position of a single-tooth implant. The first registration data (postsurgical implant position data) and the presurgical CBCT volume data can be superimposed to enable visualization of the 3D position of the inserted implant and its relationships with the surrounding anatomical structures.

Derksen et al. [21] evaluated the accuracy of computer-guided implant surgery using tooth-supported templates in 66 patients who received 145-Straumann tissue level implants. They used postsurgical intraoral scanning, rather than a postsurgical CBCT scan, and they imported the data into coDiagnostiX software (coDiagnostiX; Dental Wings GmbH). The “Treatment Evaluation Tool” component of the software was used to evaluate the accuracies of the surgical templates. The study revealed mean deviation values of 0.75 mm, 1.06 mm, and 2.72° for the implant entry point, apex point, and angulation, respectively; these were similar to our results obtained using digital registration (0.672 mm, 1.160 mm, and 2.700° for entry point, apex point, and angle, respectively). Derksen et al. [21] reported that three patients (treated with six implants) underwent a second CBCT scan for other reasons, which allowed analyses of the accuracy of guided surgery using postsurgical CBCT. The results were comparable with the intraoral scan findings; the maximum reported difference in deviation between the two methods was 0.20 mm. These results are consistent with the resolution of CBCT (voxel size: 0.2 mm) and are similar to the maximum tolerated difference in our study. Derksen et al. [21] suggested that additional studies are needed to confirm that the results of the digital registration method are similar to the results of conventional postsurgical CBCT. Our analysis of accuracy evaluation showed that digital registration produced results similar to the conventional radiographic method findings.

Using a paired t-test analysis, Tang et al. [23] found no significant difference in terms of accuracy evaluation between the digital registration and radiographic methods in 19 patients with 32 implants (*p* > 0.05). The paired t-test analysis was performed to identify any significant differences between the methods on average. *P* > 0.05 just indicated that the current evidence could not prove the mean difference between the two methods was not 0; however, the results of that assessment could not determine the agreement between the two methods. Zhou et al. [28] measured the deviation between implant positions determined by a surface scanner and by postsurgical CBCT in 10 resin models with 40 implants. The study showed that the mean deviation values between the two methods at the entry point, apex point, and angle were 0.25 mm, 0.28 mm, and 0.68°, respectively. These mean deviation values were higher than the values in our study (0.032 mm, 0.006 mm, and 0.139° for entry point, apex point, and angle, respectively). These discrepancies may have arisen because Zhou et al. used models in which multiple teeth had been lost, while we used models in which a single tooth had been lost. Furthermore, in contrast to our study, Tang et al. and Zhou et al. did not use surgical templates. Franchina et al. [29] made a comparison of postsurgical optical scanning versus postsurgical CBCT in assessing the accuracy between planned and achieved implants. Ninety implants in fifteen resin models were digitally planned and then placed following three different approaches: template guided free hand, static computer aided implantology and dynamic computer aided implantology in their study. They also found no statistically significant mean difference (*p* > 0.05) between optical scanning and CBCT for the implant accuracy assessment using an independent-samples t-test analysis.

Paired t-test and simple correlation analyses are not appropriate for evaluating the agreement of results obtained through different methods [30]. The appropriate methods for such an evaluation include ICC, Bland–Altman plots, and ATE/LER zones (allowable total error/limits for erroneous result zones) [34, 35]. In the present study, we used ICC analysis and Bland–Altman plots. ICCs are useful for evaluating the agreement of results measured by different methods or observers. Larger ICCs are associated with smaller variation caused by systematic and random errors. ICCs range from 0 to 1; ICCs > 0.75, 0.40–0.75, and < 0.4 indicate good, moderate, and poor agreement, respectively [30]. The ICCs between the digital registration and conventional radiographic methods for the entry point, apex point, and angle were 0.986, 0.993, and 0.968, respectively, indicating satisfactory agreement between the two methods. Bland–Altman analysis, originally proposed by Bland and Altman in 1986 [34], involves the use of the mean and difference between two groups of data to construct a scatter plot where the mean is shown on the horizontal axis and the difference is shown on the vertical axis. The scatter plot is used to calculate the mean difference and LoA for the difference value (i.e., − 1.96 SD to + 1.96 SD). If the 95% distribution range of the difference is within the LoA or the LoA is within the range of the clinically acceptable threshold value (i.e., maximum tolerated difference), the agreement between the two groups is good [32, 35, 36]. CBCT images are acquired at a voxel resolution of 0.2 mm; therefore, a precision better than 0.2 mm cannot be achieved [20, 27]. In the present study, the maximum tolerated difference for the measurements of the entry and apex points was 0.200 mm, while the maximum tolerated difference for the angle was 0.881°. The LoAs of the difference values for the three measurements were all within the maximum tolerated difference range, indicating good agreement between the two methods.

Our results indicated the digital registration method could be used for accuracy evaluation of single-tooth implant surgery using tooth-supported surgical templates and acrylic resin models. Our model analyses were conducted using an ideal environment; therefore, further clinical studies are necessary to confirm the effectiveness of the new method. The digital registration method can only be performed for partially edentulous individuals using tooth-supported templates. Because dentition constitutes an optical and radiographic marker, alignment was conducted based on the natural teeth [37, 38]. This method may not be appropriate for the mucosa- and bone-supported surgical templates that are used in edentulous patients due to their lack of natural teeth [39]. Further investigations are needed to determine whether other alignment reference points (e.g., fixation pins and temporary implants) can be used for the digital registration method.

## Conclusions

The present in vitro study showed that digital registration and conventional radiographic methods have good agreement in terms of evaluating the accuracy of implant positioning using tooth-supported surgical templates. The digital registration method may be useful for postsurgical evaluation of single-tooth implant accuracy, although further clinical studies are needed to confirm our results.

## Data Availability

All essential data is presented in the manuscript. The step-by-step datasets and images during the current research are available from the corresponding author on reasonable request.

## Abbreviations

CBCT: Cone-Beam Computed Tomography
3D: Three-Dimensional
2D: Two-Dimensional
STL: Standard Tessellation Language
DICOM: Digital Imaging and Communications in Medicine
FOV: Field-of-View
ICCs: Interclass Correlation Coefficients
LoA: Limits of Agreement
SD: Standard Deviation
